# Rurality and Self-Reported Health in Women with a History of Intimate Partner Violence

**DOI:** 10.1371/journal.pone.0162380

**Published:** 2016-09-13

**Authors:** Gina Dillon, Rafat Hussain, Deborah Loxton, Asad Khan

**Affiliations:** 1 School of Rural Medicine, University of New England, Armidale, NSW, 2351, Australia; 2 ANU School of Medicine & Research School of Population Health, Australian National University, Canberra, ACT, 0200, Australia; 3 Research Centre for Generational Health and Ageing, University of Newcastle, Callaghan, NSW, 2308, Australia; 4 School of Health and Rehabilitation Services, The University of Queensland, Brisbane, QLD, 4072, Australia; University of Westminster, UNITED KINGDOM

## Abstract

**Objective:**

To investigate differences in self-reported health among Australian women with a history of intimate partner violence (IPV) in relation to rurality of residence.

**Methods:**

Data were drawn from six survey waves of the Australian Longitudinal Study on Women’s Health 1973–78 birth cohort. Self-reported general and mental health scores derived from the SF-36 scale were compared for women with a history of IPV living in metropolitan, regional and rural areas. Multivariable generalised estimating equations were constructed adjusting for income hardship, number of children, education, social support, age and marital status.

**Results:**

Women with a history of IPV living in regional and rural areas had no significant differences in self-reported general health scores compared to their metropolitan counterparts. Rural women affected by IPV had slightly better self-reported mental health than equivalent women living in metropolitan or regional areas. The socio-demographic factors with the strongest association with self-reported health were income, education, social support, and number of children.

**Conclusions:**

Women in regional and rural areas were no more disadvantaged, in terms of self-reported general health or mental health, than IPV affected women living in major cities in Australia.

## Introduction

The detrimental effects of intimate partner violence (IPV), in terms of negative mental and physical health outcomes, has been reported in clinical and community-based studies across a wide range of countries [1]. IPV has been reported to be associated with both acute and chronic physical health conditions such as: gynaecological problems, musculoskeletal injury, chronic pain, reduced levels of physical functioning and lower self-perceived levels of physical health [2, 3, 4]. IPV has also been shown to be associated with a range of mental health conditions including depression, anxiety, post-traumatic stress disorder, substance abuse, sleep disorders, self-harm and suicide [5, 6, 7]. The negative impact of IPV on health can persist for years after the abuse has ceased as evidenced by chronic physical conditions and ongoing mental health problems in women who have ever lived with a violent partner [8, 9].

The extent to which rurality of residence influences the prevalence and severity of IPV in the Australian context is still an under-researched topic. However researchers have identified characteristics of rural living that could potentially contribute to women’s vulnerability to IPV and restrict women’s capacity to exit violent relationships including: isolation [10, 11], lower levels of police staffing and large areas of police jurisdiction [12], higher alcohol consumption rates [13, 14], lack of anonymity in rural communities [15], and reduced access to IPV services [15, 16]. A recent investigation into the lifetime prevalence of IPV in Australia found significantly higher rates in rural and regional areas compared to metropolitan areas [17].

Within Australia it is recognised that there are lower levels of health service provision outside of metropolitan areas [18, 19]. Given this fact, along with reduced availability of IPV specific services, does it then follow that women with a history of IPV who reside outside of metropolitan areas have a greater health disadvantage due to exposure to IPV than do women from metropolitan areas? This area of enquiry, relating to metropolitan versus non-metropolitan residence and health of women with a history of IPV, is a distinct gap in the Australian IPV literature.

This study investigates the role of rurality on the association between exposure to IPV and self-reported health in a cohort of community-dwelling Australian women. Given the potential for multiple health disadvantages, it is hypothesised that women from rural and regional areas who have experienced IPV will experience poorer health than their metropolitan counterparts.

## Methods

### Study population

This study uses self-reported data from the Australian Longitudinal Study on Women’s Health (ALSWH), a nationally-representative longitudinal study of health, lifestyle and demographic factors of Australian women [20]. ALSWH participants were randomly selected from the Australian national health insurance (Medicare) database with deliberate oversampling in rural and remote areas, and the recruitment process and survey procedures for the ALSWH are detailed elsewhere [21, 22]. The current study includes women from the ALSWH 1973–78 birth cohort (n = 14,247) who reported having experienced a violent relationship with a partner or spouse. Data are drawn the first six survey waves for this ALSWH cohort, spanning the years 1996–2012. This study was approved by the Human Research Ethics Committee of the University of New England, approval number HE 12–125, and was approved by the ALSWH as part of Project A411.

Project A411 is the number of the current project titled “Rurality, vulnerability and intimate partner violence”. Project A411 utilises data **only** from the young cohort in relation to IPV. A synopsis of A411 is available on ALSWH website: http://www.alswh.org.au/substudies-and-analyses/analyses?projectid=A411study. All publications arising from project A411 will be limited to the scope of the study and based on analyses of data from the ALSWH young cohort only i.e., it has not and will not include any publications based on analyses of ALSWH data of mid-aged cohort (1946–51 birth cohort). The ALSWH mid-aged cohort study also includes questions on IPV; and some of the sub-studies and papers have explored issues associated with IPV in midlife. Full details of all sub-studies and secondary analysis using ALSWH data is available on http://www.alswh.org.au/substudies-and-analyses.

### Variables and measures

#### Measures of Health

Self-reported health was measured using the Medical Outcomes Study 36-item Short-Form (SF-36) Health Survey. The SF-36 is a validated measure of self-reported health consisting of 36 questions related to eight different health domains, namely: general health, physical functioning, role physical, bodily pain, vitality, social functioning, mental health, and role emotional [23].

Within this study, scores from the general health (GH) and mental health (MH) domains of the SF-36 were used. Self-reported scores for GH were derived from the General Health subscale of the SF-36 and consists of accumulated scores for the 5 general health subscale items as totalled and provided by the ALSWH as part of the dataset. Self-reported MH scores were taken from the Mental Health subscale of SF-36. The subscale scores consist of accumulated scores for the 5 mental health subscale items as totalled and provided by the ALSWH as part of the dataset. Both of these health scales range from 0–100 with higher scores indicating better health.

#### Measure of IPV

Lifetime exposure to IPV was determined via yes/no responses to the question “Have you ever been in a violent relationship with a partner/spouse?” If a woman answered yes to this question at a given survey wave, or at any previous surveys, they were recorded as having a lifetime experience of IPV.

#### Rurality of residence

The rurality of residence for each participant was recorded using the Australian Standard Geographical Classification (ASGC) categories, namely: major cities, inner regional, outer regional, remote and very remote. The ASGC is recognised as a nationally standardised measure of geographic remoteness incorporating aspects of distance and access to services [24]. Within this study the ASGC classes were consolidated to three categories of: major cities, inner regional and rural regions. The rural category was formed by combining the ASGC categories of outer regional, remote and very remote areas.

#### Socio-demographic variables

All multivariable models were adjusted for the following socio-demographic variables: Marital status, classified as: currently in a married or de facto relationship; single/never married; separated/divorced or widowed. The highest education level completed by participants at each survey was categorised as: less than completion of secondary school (Year 12); completion of secondary school; trade apprenticeship, certificate or diploma; a bachelor or higher university degree. Ability to manage on available income was categorised as: not too bad/easy; or impossible/difficult. Number of children in the household indicates the total number of children, whether the respondent’s children, children from a partner or spouse, or foster children, categorised as: no children; one child; two children; three or more children. Age (years) was recorded at each survey wave.

Availability of social support was measured via the Medical Outcomes Study Social Support Survey [25] and dichotomised as social support available: most/all of the time; or none/little/some of the time. Investigation of the distribution of the MOS-SSS scores from survey wave S4-S6 showed a skewed distribution, with more frequent availability of support predominating. This skewed distribution has been reported by the scale developers [25]. For our data, at S4, the median response (50^th^ percentile) for 14 of the 19 items on the MOS-SSS was 4, which is equivalent to ‘most of the time’. The remaining five items had a median response of 5, ‘representing ‘all of the time’. Given the skewed distribution, it was decided (based on advice of a statistical expert well-versed with ALSWH data), to dichotomise the social support responses around the median score to become a binary categorisation of social support being available ‘none/little/some of the time’ or ‘most/all of the time’. This scheme of categorisation was then applied to the social support responses from data from waves S1-S3 in order to have a consistent measure of social support across all six survey waves.

### Analysis

Generalised estimating equations (GEE) were used to investigate the longitudinal associations between self-reported health, rurality of residence and additional socio-demographic factors. GEE is a longitudinal modelling technique allowing estimation of population averaged effects while accounting for within-subject correlation from repeated measuring of the same individuals [26]. All GEE modelling was conducted using Stata12 [27] with the *xtgee* command specified as: family (gaussian), link (identity) and corr(ar1).

Analyses for general health and mental health were conducted separately. In the general health model, SF-36 mental health subscale scores were included as an additional explanatory variable. Likewise, in the mental health model, the corresponding general health subscale scores were included as an explanatory variable. Initially each explanatory variable (rurality of residence, marital status, education, income, number of children, social support, age, mental/general health score) were investigated to evaluate their unadjusted associations with self-reported health. Variables found to be associated with self-reported health (p ≤ 0.05) were included within the multivariable model.

## Results

The reported lifetime IPV prevalence within the ALSWH 1973–78 cohort increased steadily across the six survey waves: 11.9% at S1; 14.8% S2; 17.4% S3; 20.1% S4; 21.2% S5 and 21.6% at S6. Table 1 shows the socio-demographic characteristics of women with a lifetime history of IPV at each of the six survey waves. The 16 year time span from S1 (1996) to S6 (2012) represents a period of significant change in the lives of the women in the study sample, with the mean age of the sample being 20.8 years at S1 and 36.3 years at S6. At S1 the majority (64%) of women were single/never married with only 32% of women being married or in a de facto relationship. At S6 the majority (72%) of women were married or in a de facto relationship and only 13% of women reported being single/never married.

**Table 1:** Socio-demographic characteristics of women with a lifetime history of IPV across six survey waves (S1-S6) of the ALSWH.

Attribute	Category	S1 (1996)		S2 (2000)		S3 (2003)		S4 (2006)		S5 (2009)		S6 (2012)	
		n^1^ = 1703		n = 1437		n = 1579		n = 1841		n = 1739		n = 1569	
		n	%	n	%	n	%	n	%	n	%	n	%
Remoteness of residence	Major cities	761	44.7	660	46.1	808	51.2	919	50.3	877	50.9	788	52.1
	Inner regional	593	34.8	472	33.0	474	30.0	562	30.8	533	30.9	456	30.2
	Rural	348	20.4	299	20.9	297	18.8	345	18.9	313	18.2	268	17.7
	**Total for Remoteness**	**1702**	**100**	**1431**	**100**	**1579**	**100**	**1826**	**100**	**1723**	**100**	**1512**	**100**
Marital status	Single/Never married	1084	64.0	680	47.5	526	33.6	417	22.8	304	17.6	199	12.9
	Married/ De facto	549	32.4	672	47.0	907	57.9	1230	67.2	1222	70.8	1107	71.7
	Separated/Divorced/Widowed	62	3.7	79	5.5	133	8.5	182	10.0	200	11.6	238	15.4
	**Total for Marital status**	**1695**	**100**	**1431**	**100**	**1566**	**100**	**1829**	**100**	**1726**	**100**	**1544**	**100**
Education	< Secondary school	568	33.5	301	22.3	288	19.1	283	15.5	225	13.5	149	9.7
	Secondary school	705	41.6	379	28.1	366	24.2	359	19.6	292	17.5	196	12.8
	Trade/Certificate/Diploma	332	19.6	365	27.0	462	30.6	640	35.0	545	32.7	547	35.7
	University degree	90	5.3	305	22.6	394	26.1	547	29.9	607	36.4	641	41.8
	**Total for Education**	**1695**	**100**	**1350**	**100**	**1510**	**100**	**1829**	**100**	**1669**	**100**	**1533**	**100**
Income Management	Impossible/difficult	1153	67.8	Not available this survey wave		917	58.3	1021	55.8	922	53.2	844	54.6
	Not too bad/easy	548	32.2			655	41.7	810	44.2	811	46.8	701	45.4
	**Total for Income**	**1701**	**100**			**1572**	**100**	**1831**	**100**	**1733**	**100**	**1545**	**100**
Social Support	Most/all the time	957	56.7	706	51.5	742	48.1	931	51.8	826	49.0	820	53.8
	None/little/some of the time	730	43.3	666	48.5	802	51.9	868	48.2	859	51.0	704	46.2
	**Total for Social support**	**1687**	**100**	**1372**	**100**	**1544**	**100**	**1799**	**100**	**1685**	**100**	**1524**	**100**
Number of children in household	None	Not available this survey wave		937	65.7	790	52.9	777	44.5	573	34.7	418	26.7
	One			272	19.1	337	22.6	406	23.3	36	22.7	298	19.1
	Two			160	11.2	244	16.3	347	19.9	417	25.2	510	32.6
	Three or more			57	4.0	123	8.2	216	12.4	287	17.4	337	21.6
	**Total for No. of children**			**1426**	**100**	**1494**	**100**	**1746**	**100**	**1653**	**100**	**1563**	**100**
Age (years)	Age range	16.9–24.4		20.6–28.5		23.6–31.1		26.6–34.6		29.6–37.3		32.0–40.1	
	Mean age	20.8		24.6		27.6		30.6		33.7		36.3	

^**1**^ Number of participants reporting a history of IPV at each ALSWH survey wave

There was a high level of ongoing education in the study participants, with the proportion of women reporting a university level of education rising from 5% at S1 to 42% at S6. As would be expected for the age of the study participants there was also a significant rise in the number of participants with children in the household. At baseline 66% of participants reported no children in their household and this proportion had dropped to 17% of women reporting no children at S6. At S1, approximately 45% of women in the study sample lived in metropolitan areas, with 35% residing in regional areas and 20% in rural areas. At S6 over half of the sample resided in metropolitan areas (52%), with 32% in regional and 18% in rural areas.

Unadjusted and adjusted models for SF-36 General Health subscale scores are presented in Table 2. In the adjusted model (GH Model 2, Table 2), the mean general health scores of women with a history of IPV did not vary significantly with rurality. This indicates that rural and regional women, with a history of IPV, did not have a disadvantage in terms of self-reported general health compared to their metropolitan counterparts.

**Table 2:** Unadjusted and adjusted GEE models of factors associated with SF-36 General Health scores in women with a history of IPV.

Self-reported general health of women with a history of IPV	Unadjusted estimates GH Model 1		Adjusted estimates GH Model 2	
Explanatory variable (reference category)	Coeff	95% CI	Coeff	95% CI
**Remoteness of residence** (Major city)				
Inner regional	1.62*	0.31–2.94	0.80	-0.67–2.28
Rural	1.69*	0.08–3.29	0.03	-1.73–1.79
**Income management** (Impossible/difficult)				
Not too bad/easy	4.08**	2.76–5.40	3.03**	2.00–4.06
**Number of Children** (No children)				
1 child	2.01**	0.77–3.24	0.93	-0.43–2.29
2 children	2.41**	0.99–3.82	1.52	-0.04–3.09
3 or more children	2.42**	0.57–4.27	1.22	-0.78–3.23
**Education** (Less than secondary school)				
Secondary school completion	0.33	-1.62–2.28	1.66	-0.67–3.99
Trade/certificate/diploma	1.64	-0.20–3.48	2.05	-0.10–4.19
University/higher degree	5.17**	3.09–7.25	4.14**	1.85–6.44
**Social support available** (Most/all the time)				
None/little/some of the time	-4.99**	-5.89 –-4.09	-2.16**	-3.22 –-1.10
**Marital status** (Married/De facto)				
Single/Never married	-2.06**	-3.12 –-0.99	0.23	-1.22–1.67
Separated/divorced/widowed	-1.33	-2.99–0.34	-0.16	-1.92–1.60
**Age**	0.45**	0.34–0.56	0.04	-0.14–0.21
**Mental Health subscale score**	0.42**	0.39–0.44	0.41**	0.38–0.44

* p ≤ 0.05

** p ≤ 0.01

Multivariable GEE analysis revealed that the factors showing the strongest association with general health scores, indicated by the largest coefficient values (GH Model 2), were the availability of social support, income management and level of education. After adjustment for other variables within GH Model 2, women who had social support that was available ‘none/little/some of the time’ had a mean general health score that was 2.16 points lower than women with social support available ‘most/all of the time’ (p ≤ 0.01; 95% CI -3.22 –-1.10). Women who found it ‘not too bad/easy’ to manage on their income had general health scores that were on average 3.03 points higher than women who found it ‘impossible/difficult’ to manage on their income (p ≤ 0.01; 95% CI 2.00–4.06). Women with a university level of education had a mean general health score that was 4.14 points higher than women who had not completed secondary school (p ≤ 0.01; 95% CI 1.85–6.44).

Unadjusted and adjusted models for SF-36 Mental Health subscale scores are given in Table 3. Multivariable GEE modelling indicated a slight effect of rurality on the mean mental health scores of women with a history of IPV. Women who lived in rural areas had a significantly higher mean mental health score than women who lived in major cities (p ≤ 0.05; 95% CI 0.26–3.18). Women from inner regional areas had no mean difference in mental health scores compared to their counterparts in major cities (MH Model 2, Table 3).

**Table 3:** Unadjusted and adjusted GEE models of factors associated with SF-36 Mental Health scores in women with a history of IPV.

Self-reported mental health of women with a history of IPV	Unadjusted estimates MH Model 1		Adjusted estimates MH Model 2	
Explanatory variable (reference category)	Coeff	95% CI	Coeff	95% CI
**Remoteness of residence** (Major city)				
Inner regional	1.08	-0.14–2.30	1.14	-0.10–2.38
Rural	1.27	-0.20–2.73	1.72*	0.26–3.18
**Income management** (Impossible/difficult)				
Not too bad/easy	4.43**	3.09–5.77	1.55**	0.58–2.53
**Number of Children** (No children)				
1 child	2.46**	1.21–3.70	2.72**	1.46–3.98
2 children	2.35**	0.98–3.72	2.45**	1.04–3.86
3 or more children	2.00*	0.25–3.76	1.54	-0.22–3.29
**Education** (Less than secondary school)				
Secondary school completion	1.67	-0.11–3.44	2.63*	0.68–4.59
Trade/certificate/diploma	2.92**	1.23–4.61	2.46**	0.67–4.25
University/higher degree	5.40**	3.59–7.22	2.92**	1.05–4.79
**Social support available** (Most/all the time)				
None/little/some of the time	-9.66**	-10.54 –-8.78	-7.34**	-8.31 –-6.37
**Marital status** (Married/De facto)				
Single/Never married	-3.71**	-4.75 –-2.68	0.33	-1.28–1.35
Separated/divorced/widowed	-3.91**	-5.56 –-2.26	-1.01	-2.63–0.60
**Age**	0.47**	0.36–0.57	0.13	-0.03–0.28
**General Health subscale score**	0.42**	0.40–0.44	0.36**	0.34–0.39

* p ≤ 0.05

** p ≤ 0.01

Within the adjusted model, the factors with the strongest association with mental health scores were social support, education and number of children resident in the household. Women with reduced social support had a mean mental health score that was 7.34 points lower (p ≤ 0.01; 95% CI -8.31 –-6.37) than women who had social support available ‘most/all the time’. Higher levels of education were associated with higher mental health scores. Women with a university level education had a mean mental health score that was 2.92 points above the mean score of women who had not completed senior secondary school education (p ≤ 0.01; 95% CI 1.05–4.79). Women who reported that it was ‘not too bad/easy’ to manage on their current income had mean mental health scores that were 1.55 points higher than women who found it ‘impossible/difficult’ to manage on their income (p ≤ 0.01; 95% CI 0.58–2.53). Having one or two children in the household was associated with mental health scores that were 2 points higher than women with no children in the household (p ≤ 0.01).

## Discussion

This study investigated differences in the self-reported health of women with a history of IPV across different categories of rurality within Australia. Contrary to our hypothesis, it was found that, amongst women with a history of IPV, those in regional and rural areas were no more disadvantaged in terms of self-reported general health or mental health compared to IPV affected women living in major cities. In fact, women with a history of IPV living in rural areas showed slightly higher levels of self-reported mental health compared to their counterparts in major cities. This is a new area of enquiry and no comparative studies from Australia were found.

Rural and regional areas of Australia are relatively under-resourced compared to metropolitan centres in terms of health services [18] and IPV related support services [16]. So, given this relative lack of services, the results from this study showing equivalent, or in some cases higher, self-reported health in non-metropolitan IPV-affected women, is perhaps surprising. But this apparent paradox of health is also evident across the general Australian population in relation to a range of health issues. In a study of health indicators across non-metropolitan areas of Australia it was reported that, compared to their counterparts in major cities, residents in non-metropolitan areas have: higher rates of mortality, injury and disability; higher engagement in health risk behaviours especially smoking and excessive alcohol consumption and increased rates of obesity, diabetes and cardiovascular disease [28]. Yet, despite these indicators of significant health differentials across non-metropolitan areas, evidence from all three age cohorts of the ALSWH indicate few remoteness-based differences in self-reported measures of physical or mental health in Australian women [29].

This seeming contradiction between self-reported perceptions of health and recorded lower levels of rural health was studied by Harvey [30] who identified that health and well-being, as defined by rural women, involves acknowledging the significance of the specific social context of living in rural areas. This social context may change the perceptions of these concepts in rural women compared to urban women. This raises an interesting area for future research, namely the concepts of well-being, resilience and coping in IPV-affected women across the range of remoteness categories in Australia, and the ways that these concepts interact with these women’s perceptions of health and specific IPV service needs.

For women who had reported a history of IPV, the availability of social support emerged as a significant factor in relation to self-reported health, particularly mental health. IPV affected women who had social support that was available ‘none/little/some of the time’ had general health scores that were 2 points lower, and mental health scores that were 7 points lower than women with more readily available social support. This result corresponds with previous findings of the role of social support in protecting against the negative impact of IPV on both physical and mental health [31, 32, 33, 34]. It has also been reported that social support was not only positively associated with women’s mental and physical health during the crisis of leaving an abusive partner, but was also an important determinant of women’s health up to 20 months after leaving [35].

Income hardship was also significantly associated with lower levels of self-reported health. Among women with a history of IPV, those women who reported that it was relatively easy to manage on their available income had higher mean mental and general health scores than IPV affected women who reported income hardship. These findings are in line with previous research which has found that abuse was more strongly associated with poor physical health symptoms at lower levels of income [36]. Income has also been identified as a protective factor in the mental health of abused women, with an absence of income hardship providing a buffer against some of the negative effects of abuse on mental health [31].

One explanation for the association between low income and poorer health is that financial deprivation may limit access to medical care, which may in turn lead to higher rates of unresolved medical problems including mental health issues and chronic physical symptoms. Within the ALSWH 1973–78 birth cohort, an investigation into the use of health services by IPV affected women found that income hardship was not associated with reduced access to health services [37]. However it is still possible that income hardship may affect the quality of medical care, or limit the ability to follow up on suggested medical care regimes due to lack of financial resources, meaning that women with more financial hardship still have reduced opportunities to gain full benefit from medical care, despite having equal access to health practitioners.

Better mental health scores for IPV affected women with one or two children in the household is an interesting result, and one which seems contrary to the notion of increased levels of stress that may occur with the responsibilities of parenting. The financial burden of parenting is particularly relevant in IPV affected, single mothers, as caring for children not only increases financial costs directly, but can also restrict employment and earning capacity of women if affordable childcare is not available [38, 39, 40]. Having children in your care restricts housing options and increases difficulties in securing suitable and affordable accommodation quickly [41]. Sharing a child with an abusive ex-partner increases the need for continued contact through custody and co-parenting arrangements which can add considerable stress to a woman’s life as well as leaving her significantly more vulnerable to re-abuse from her ex-partner [42, 43].

However, there are also positive aspects of parenting to be considered, including the mutual care and support from the mother-child relationship which can provide a buffer from the negative effects of abuse and an increased sense of purpose and self-esteem as a parent [44, 45, 46]. The presence of children can also be a positive factor for social interaction, allowing mothers of younger children to meet [47], potentially lessening social isolation and therefore enhancing mental health.

A search of the literature failed to find any studies on the association between children in the household and validated measures of mental health in IPV affected women. There have however been studies in the general population which have indicated that mothers have better self-reported general and mental health [48] and greater life satisfaction [49] than childless women. The results of the current study are in line with these findings, and suggest that there are positive benefits of motherhood for mental health in IPV affected women, despite the additional stress that child rearing may present. The lack of published findings regarding the association of children with mental health in IPV affected women highlights an area of needed research.

In the current study, IPV affected women with higher levels of education had higher self-reported general and mental health. These results align with previous studies indicating that abused women with higher levels of education were less likely to report depression and anxiety compared to those with lower education [31]. Better levels of education could indirectly benefit health by enabling greater potential earning capacity, better employment opportunities, increased economic autonomy, higher levels of self-esteem [50], better social networks and higher social capital [51]. Women with higher levels of education are also more likely to access services in response to IPV [52, 53] which could positively influence health outcomes.

The current study indicated that IPV affected women with poorer levels of education, reduced social support and increased levels of income hardship had lower levels of self-reported health. These results identify possible areas of intervention that could be used to reduce the impact of IPV on women’s health. Social support systems can be enhanced via advocacy programs [47] and through programs to help educate family and friends of victims about responses that can help encourage the pursuit of support through formal services including domestic violence agencies, healthcare and legal services [54]. Issues of income hardship can be addressed via subsidised housing, provision of free medical care, including counselling services. Longer term benefits could be gained from investment in targeted interventions that allow women to become financially independent and improve their employment prospects, for example subsidised childcare; unemployment compensation if required to leave employment due to IPV related harassment or relocation; and more flexible workplace agreements for single mothers [55].

A limitation of the current study is that the data related to IPV within the ALSWH surveys did not differentiate between recent and more temporally distant abuse, thus the influence of the recency of abuse on health outcomes could not be determined. It is also worth noting that the single item measure of abuse that required women to identify their partner as ‘violent’ generates a conservative estimate of IPV. Future research that includes more detailed measures of IPV, including behavioural items, would be valuable in determining the prevalence and impact of particular types of abuse by area. The major strengths of this study include the use of a sample of women from a national population-based cohort study, a large sample size, the ability to analyse the results by rurality of residence, and the use of widely used and validated health scales (SF-36).

## Conclusion

This study is the first Australian longitudinal investigation into the role of rurality on self-reported health in women who have experienced IPV. It was found that IPV affected women in regional and rural areas were no more disadvantaged, in terms of self-reported general health or mental health, than those living in major cities. These results are surprising, given that regional and rural areas of Australia are recognised as having poorer access to medical services and IPV related support services compared to metropolitan areas.

Several socio-demographic factors were identified as being associated with lower levels of self-reported health. Women reporting income hardship and low levels of social support reported the poorest levels of general and mental health. Interventions that help provide additional social support and help foster financial independence in IPV affected women may reduce the burden of poor health in these women.
